# Poultry trading behaviours in Vietnamese live bird markets as risk factors for avian influenza infection in chickens

**DOI:** 10.1111/tbed.13308

**Published:** 2019-08-09

**Authors:** Joshua E. Sealy, Guillaume Fournie, Pham Hong Trang, Nguyen Hoang Dang, Jean‐Remy Sadeyen, To Long Thanh, H. Rogier van Doorn, Juliet E. Bryant, Munir Iqbal

**Affiliations:** ^1^ The Pirbright Institute Pirbright Woking UK; ^2^ The Royal Veterinary College London UK; ^3^ The National Centre for Veterinary Diagnostics Hanoi Vietnam; ^4^ The Department of Animal Health Hanoi Vietnam; ^5^ Oxford University Clinical Research Unit‐Hanoi Hanoi Vietnam; ^6^ Laboratory of Emerging Pathogens Fondation Mérieux Lyon France

**Keywords:** Avian influenza, H9N2, live bird markets, phylogenetics, poultry trading habits, risk factors, Vietnam

## Abstract

Vietnamese poultry are host to co‐circulating subtypes of avian influenza viruses, including H5N1 and H9N2, which pose a great risk to poultry productivity and to human health. AIVs circulate throughout the poultry trade network in Vietnam, with live bird markets being an integral component to this network. Traders at LBMs exhibit a variety of trading practices, which may influence the transmission of AIVs. We identified trading practices that impacted on AIV prevalence in chickens marketed in northern Vietnamese LBMs. We generated sequencing data for 31 H9N2 and two H5N6 viruses. Viruses isolated in the same LBM or from chickens sourced from the same province were genetically closer than viruses isolated in different LBMs or from chickens sourced in different provinces. The position of a vendor in the trading network impacted on their odds of having AIV‐infected chickens. Being a retailer and purchasing chickens from middlemen was associated with increased odds of infection, whereas odds decreased if vendors purchased chickens directly from large farms. Odds of infection were also higher for vendors having a greater volume of ducks unsold per day. These results indicate how the spread of AIVs is influenced by the structure of the live poultry trading network.

## 1 INTRODUCTION

Avian influenza viruses (AIVs), including H5N1 and H9N2, are endemic within poultry production systems in many low‐ and middle‐income countries (LMICs) and pose a significant threat to food security and to human health. Zoonotic outbreaks incur a severe economic burden through patient medical costs and stamping out programmes that can run into billions of dollars, while persistence of AIVs in poultry rearing systems causes poultry morbidity and mortality (Alexander, 2007; Otte, Hinrichs, Hinrichs, Rushton, Roland‐Holst, & Zilberman, 2008; Qi et al., 2014). Humans are immunologically naïve to AIVs; however, sporadic human cases are reported each year from countries with high levels of AIV endemicity, and although sustained transmission in humans does not occur, there is a clear ongoing threat of pandemic emergence for these viruses (Uyeki et al., 2002).

Poultry production and trade in LMICs are heterogeneous, with different species being brought together from various size farming systems, often without robust biosecurity (Fournié et al., 2016; Webster, 2004). Live bird markets (LBMs) are a traditional aspect of these systems that facilitate the storage and sale of live poultry including chickens, ducks, quail and pigeons. As a consequence, LBMs play a significant role in the maintenance and spread of AIVs and thus pose a zoonotic risk to poultry workers and consumers, and to temporary workers enlisted during stamping out programmes (Bridges et al., 2002; Mounts et al., 1999). LBMs have been a primary target for AIV control strategies; during a zoonotic outbreak of H7N9 in China in 2013, closure of LBMs was shown to be remarkably effective in reducing the risk of human infection by up to 99% (Yu et al., 2014). Control strategies in LBMs have also been shown to significantly reduce AIV detection in chickens: the most effective strategies include monthly rest days that involve routine market closure followed by slaughter of unsold poultry, a ban to the sale of live quail and a ban to overnight storage of live poultry (Kung et al., 2003; Lau et al., 2007; Leung et al., 2012). However, although rest days are effective at breaking the viral amplification cycle in LBMs, they do not prevent reintroduction of virus. Indeed, rest days/nights are an important component of long‐term AIV control but are not sufficient alone to eliminate infection (Kung et al., 2003). Furthermore, risk factor studies in LBMs have shown that having a greater variety of poultry species, including ducks being sold alongside other species, having poor sanitary conditions, storing poultry in floor pens instead of cages and having ≥1 wholesaler trading in LBMs, all increase the odds of having AIV‐infected poultry and/or having AIV‐contaminated environments (Kim et al., 2018; Kirunda et al., 2015; Santhia et al., 2009; Sayeed et al., 2017; Wang et al., 2018).

Vietnam has enzootic H5N1 and H9N2 and is at risk of incursion by H7N9 due to a shared border with China (Thuy et al., 2016). Poultry traders are an integral component of poultry production in Vietnam. They transport poultry from farms to LBMs, shaping a live poultry trading network through which AIVs may spread. Traders’ practices may thus impact on the likelihood of introducing AIVs in LBMs and also facilitate the amplification of AIV circulation within marketed chicken populations (Fournié et al., 2012). However, a quantitative assessment of the association between poultry management practices and AIV prevalence in marketed chickens is lacking. To address this gap, AIV infection status of chickens in Vietnamese LBMs was assessed and the practices of traders offering them for sale characterized. This allowed us to assess the extent to which those practices may impact on the risk of viral circulation in LBMs.

## 2 METHODS

### 2.1 Sample collection

Eight live bird markets (six retail and two wholesale) in four provinces of northern Vietnam were included in our study, which was conducted between 2 October 2017 and 3 December 2017. Markets were selected if they had previously been confirmed positive for AIV in chickens within the past 12 months according to FAO‐supported surveillance conducted by the National Centre for Veterinary Diagnostics (NCVD) and the Department of Animal Health (DAH, Hanoi). Markets were also selected if they were open seven days per week, facilitated the trade of live chickens, ducks and pigeons, and had more than 10 poultry traders operating in them. There were no quail (live or dead) at any of the LBMs, although this was not by design. Each LBM was sampled daily for seven consecutive days. On each day the LBM was sampled, the first 10 traders to arrive who contained at least five chickens in their flocks were recruited for the study and oropharyngeal swabs were collected from 5 chickens in each of their respective flocks, which were then pooled together. Selected traders were then asked about their recent trading practices in a closed‐ended questionnaire (Supplementary information Data S1). A total of 493 pools were collected from 2,465 chickens, and swabs were pooled in 2 ml virus transport medium (VTM) (Eagle's minimum essential medium supplemented with gentamicin, penicillin, streptomycin, bovine serum albumin, fungizol and HEPES solution) per trader and linked to traders and their questionnaire responses (on some days, less than 10 traders were sampled). Of the 493 sampled poultry traders, seven were removed during univariable and multivariable analysis due to incomplete feedback. Environmental swabs were taken from three discrete areas of markets to determine the level of influenza virus A contamination of LBM environments. These discrete areas represented different poultry‐related work activities which had previously been recommended to be included in routine monitoring and surveillance programmes for avian influenza viruses in LBMs (Indriani et al., 2010): (a) slaughter area including equipment used for slaughtering birds, (b) waste area including bins and containers used for disposing of bird waste such as feathers and (c) poultry stall including cages and the vicinity where birds were stored during LBM trading hours. Three swab samples were taken from each area and pooled each day (three separate pools representing three sampled areas generated per day). All pooled swabs were maintained in cold chain for transportation to NCVD, Hanoi, where they were stored at −70°C until further processing.

### 2.2 Sample screening and virus isolation

Virus RNA was extracted from pooled swab VTM using the QIAamp Viral RNA Mini kit (Qiagen) as per manufacturer's protocol. Viral RNA was screened for influenza virus A by RT‐qPCR using primers for M gene detection (M‐5 forward: AGATGAGYCTTCTAACCGAGGTCG; M‐5 reverse: TGCAAANACATCYTCAAGTCTCTG; Probe: FAM‐TCAGGCCCCCTCAAAGCCGA‐BHQ1). The threshold for influenza virus‐positive samples was Ct < 35. Subtyping using H5, H7 and H9‐specific primers was conducted on M gene‐positive samples with Ct < 26, and the threshold for subtype positivity was Ct < 38. Thermal cycling conditions were as follows: 50°C for 15 min, 95°C for 2 min, then 40 cycles of 95°C for 10 s and 60°C for 30 s.

To ensure we could obtain sequencing data from our samples, we employed next‐generation sequencing (NGS) on PCR products generated from viral RNA taken directly from pooled swab samples (Passage 0) and from infected allantoic fluid (Passage 1). Embryonated hens’ eggs were inoculated with VTM from pooled swabs which had a Ct < 27 for H9 or any Ct for H5 and “unknown” subtype‐positive samples. Allantoic fluid was harvested after 48 hr of incubation and confirmed for influenza virus A by haemagglutination (HA) assay. Viral RNA was extracted from allantoic fluid as above. In total, 50 samples meeting the above criteria were passaged in eggs, and this yielded 34 samples positive for HA activity. Passage 0 and Passage 1 samples were both subjected to NGS, and where possible, sequencing data for Passage 0 were used in phylogenetic analysis. Sequencing data were generated for a total of 33 viruses (31 H9N2 and 2 H5N6).

### 2.3 Next‐generation sequencing

Multisegment RT‐PCR was conducted on viral RNA yielded directly from the VTM of pooled swabs and from inoculated allantoic fluid. Briefly, this involved multigene amplification using the SuperScript^TM^ III One‐Step RT‐PCR kit *(*Life Technologies) and the MBTUni12/13 universal primer set with specificity towards the conserved untranslated regions (UTRs) of each influenza virus gene (Zhou et al., 2009). These PCR products were used to generate DNA libraries using the Nextera XT DNA Library Prep kit (Illumina), and an Illumina MiSeq was used to sequence pooled DNA libraries. The resultant sequencing reads were assembled via templated assembly in SeqMan NGen and consensus‐level sequences generated in SeqMan Pro (DNASTAR). Sequences were uploaded to the NCBI database with accession numbers: MN176637‐MN176652, MN176660‐MN176690, MN176731‐MN176746, MN176999‐MN177029, MN177055‐MN177085, MN177086‐MN177116, MN177518‐MN177548 and MN177635‐MN177665.

### 2.4 Phylogenetic analysis

Alignment and analysis of nucleotide and deduced amino acid sequences were conducted using MEGA7 (Kumar, Stecher, Stecher, & Tamura, 2016). Neighbour‐joining trees with 1,000 bootstrap replicates were also generated using MEGA7, and reference sequences for use in analysis alongside sequencing generated in this study were downloaded from the NCBI and GISAID databases.

### 2.5 Statistical analysis

All statistical analyses were conducted using RStudio 2016. Data from hard copy questionnaires were entered into a Microsoft Access database. Logistic regressions were used to conduct univariable analysis of explanatory variables where Influenza virus A infection status of each pool of 5 chicken swabs was used as the response variable. Explanatory variables with *p < .05* were explored for collinearity by computing VIF values with the *vif()* function in the “car” package. All variables with *p < .05* from univariable analysis had VIF < 5 so were kept for subsequent stepwise variable selection. A final model of explanatory variables with LBMs as random effects was used in multivariable analysis. Final selection of explanatory variables was conducted by backward stepwise variable selection in R. Mantel tests were conducted in the R package, “ecodist”, where virus isolates with whole‐genome sequencing data were included (Mantel, 1967). For each of the virus isolates, the ORF of each gene segment was concatenated and a dissimilarity matrix, or genetic distance matrix, was constructed from the pairwise nucleotide differences in MEGA (Kumar et al., 2016). Additional dissimilarity matrices were also constructed from the explanatory variables of same dimension as the genetic distance matrix, and related to the characteristics of the poultry from which the viruses were isolated. We refer to them as sample characteristic matrices M. For any of those matrices, an element m_ij_ = 1 if strains i and j are from samples with the same characteristic (e.g. poultry sold in the same market, poultry originating from the same type of premise, farm or market, and poultry originating from the same province), if not, mij = 0 (e.g. poultry sold in different markets, poultry originating from different types of premises and from different provinces).

## 3 RESULTS

### 3.1 Influenza virus A prevalence in live bird markets

Of 493 pooled oropharyngeal swabs from chickens, 169 (34%) were confirmed positive for influenza virus A by reverse transcription‐qPCR (RT‐qPCR) targeting the matrix (M) gene (Ct < 35) (Table 1). Subtyping of M gene positives with a Ct < 26 (*n* = 113) showed that 96% (*n* = 109) of pools had H9, 14.1% (*n* = 16) had H5, 12.3% (*n* = 14) had H9 and H5 co‐detected and 1.7% (*n* = 2) could not be subtyped. There were no samples with detectable H7 influenza virus. Influenza virus prevalence varied greatly between LBMs with the two wholesale LBMs having the least amount of detectable influenza virus (Table 1). Of the 154 pooled environmental swabs, 70 (45%) were confirmed positive for influenza virus A. The proportion of positive pooled samples was similar across the different market areas that were sampled: poultry stall area (38%, *n* = 27); waste area (34%, *n* = 24); and slaughter area (27%, *n* = 19) (birds were not slaughtered in one LBM, for which it was not therefore possible to collect swabs samples for slaughter or waste sites).

**Table 1:** Influenza virus A prevalence in selected LBMs

LBM	Province	Type	M gene Ct < 35 (%)	H9 gene Ct < 38	H5 gene Ct < 38	Subtype undetermined
Thi Cau	Bac Ninh	Retail	21 (12.4)	15	2	0
Do	Bac Ninh	Retail	31 (18.3)	18	3	2
Ga	Bac Ninh	Wholesale	6 (3.5)	3	1	0
Ha Vy	Hanoi	Wholesale	2 (1.1)	0	0	0
Ngu Hiep	Hanoi	Retail	46 (27.2)	26	7	0
Tuc Duyen	Thai Nguyen	Retail	11 (6.5)	7	0	0
Ngan	Hung Yen	Retail	17 (10)	14	0	0
Pho Hien	Hung Yen	Retail	35 (20.7)	26	3	0
		Total	169	109	16	2

Note

Data represent pooled oropharyngeal swabs. From the 169 pools positive for influenza virus A, 113 were subtyped.

Fourteen samples were positive for both H5 and H9.

Subtype undetermined refers to samples that were positive for M gene but negative for H5, H7 and H9 subtype by RT‐qPCR.

No sequencing data were available for these samples.

### 3.2 Phylogenetic analysis

**Figure 1:**
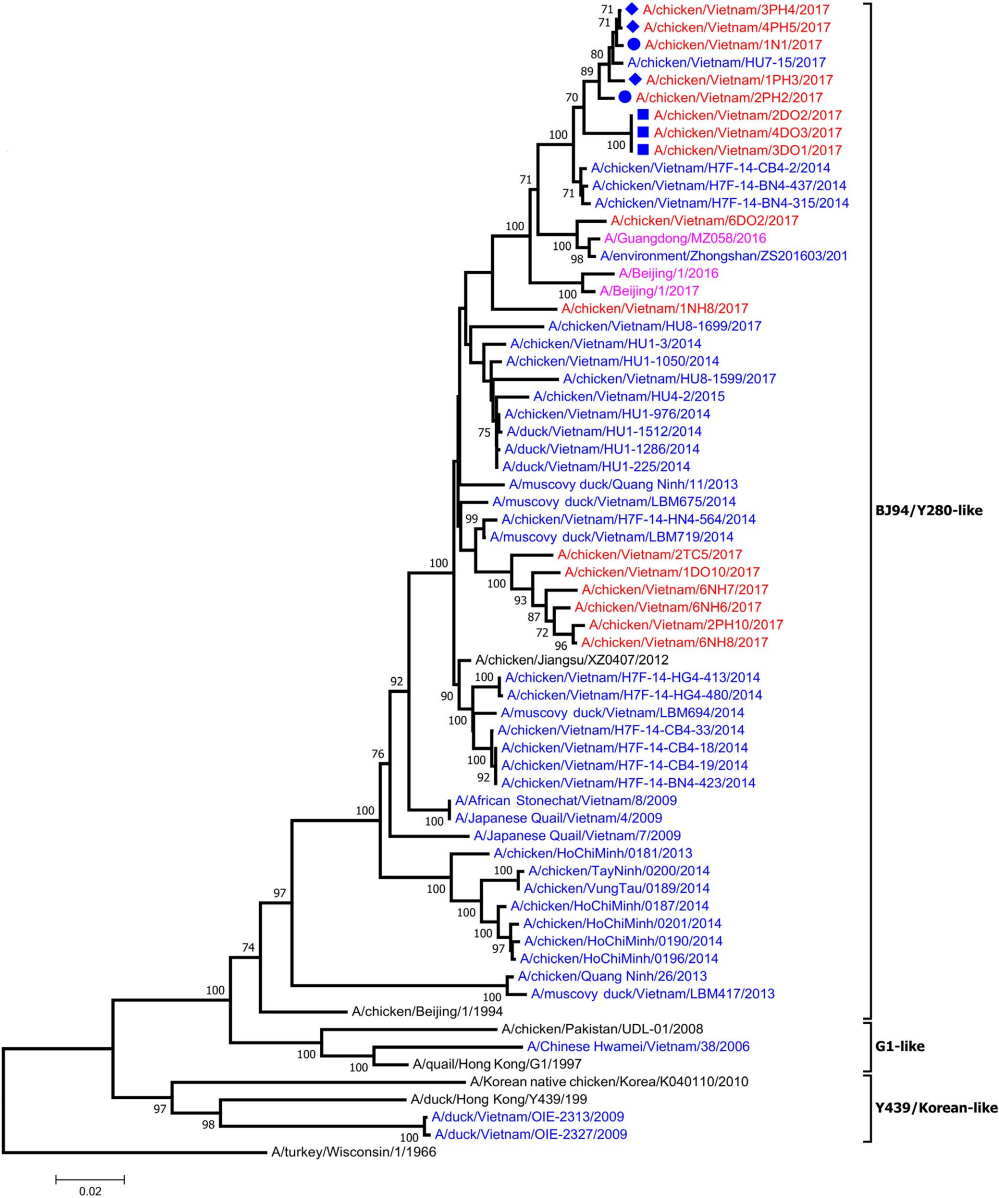
Phylogenetic tree of H9 HA. Neighbour‐joining tree representing phylogeny of H9HA sequences generated in this study; reference strains from NCBI and GISAID databases are included for comparison. Trees were formed with 1,000 bootstrap replicates, and bootstrap values <70 are not shown. In blue are reference Vietnam strains, in red are strains from this study, in black are non‐Vietnam strains, and in fuchsia are recent (2016–2018) human isolates. Blue markers represent sub‐genotypes which contain more than one virus sequenced in this study: filled circle is VN2, filled diamond is VN4, and filled square is VN5 [Colour figure can be viewed at http://wileyonlinelibrary.com]

Next‐generation sequencing (NGS) of M gene‐positive samples yielded whole‐genome sequence data for 12 H9N2 viruses and partial genomes for 19 H9N2 and 2 H5N6 viruses. H9N2 viruses sequenced in this study were most closely related to previously sequenced H9N2 viruses from Vietnam (Thuy et al., 2016) (Figure 1 and Figure S1). For example, BLASTn analysis of the PB1 gene of A/chicken/Vietnam/1DO10/2017 from this study was most closely related to A/chicken/Vietnam/H7F‐BG4‐383 with nucleotide homology of 98%. These viruses retained the G57‐like genotype, a prevalent genotype of H9N2 viruses in China known to be donors of all six internal genes to zoonotic H7N9 and H10N8 viruses (Pu et al., 2015).

**Figure 2:**
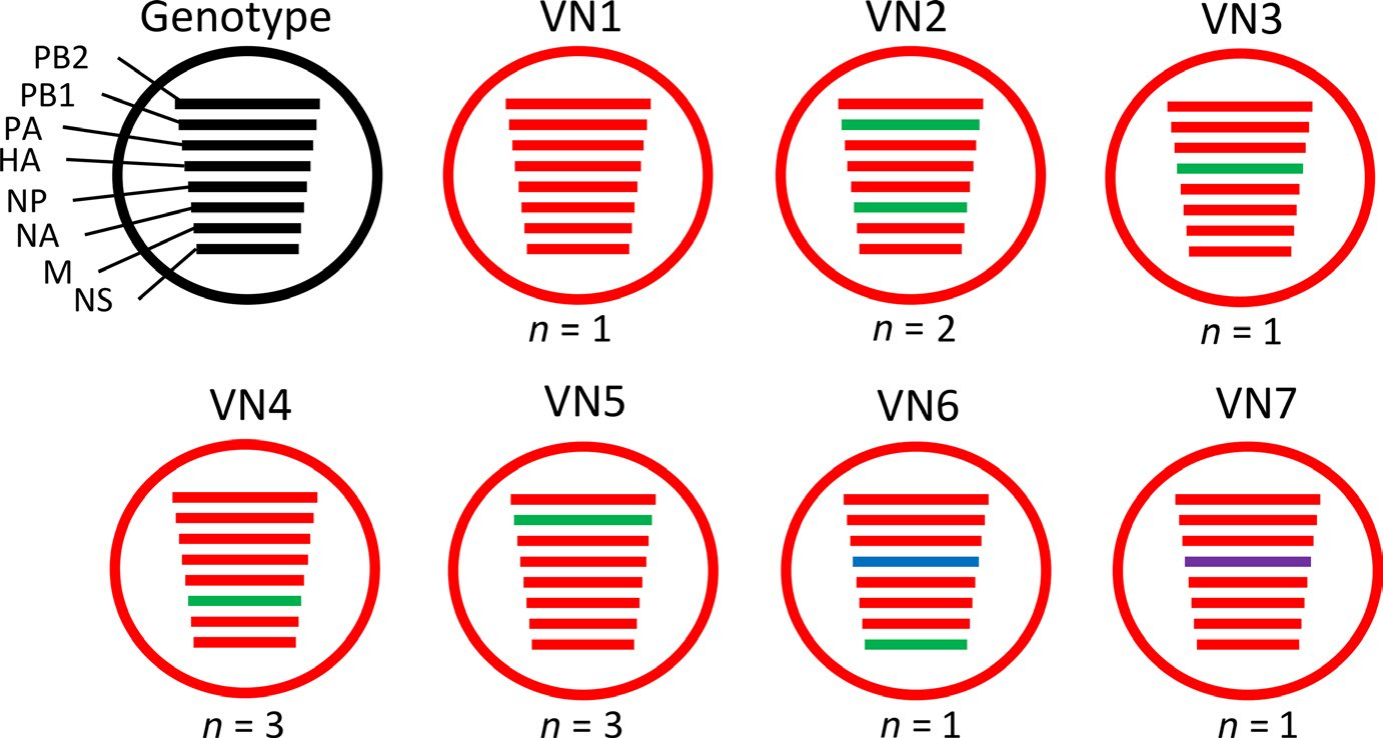
Sub‐genotypes of H9N2 viruses. Viruses with full‐genome sequencing data could be assigned to seven sub‐genotypes and are represented here. Gene segments of a different colour indicate >2% nucleotide difference [Colour figure can be viewed at http://wileyonlinelibrary.com]

We assessed whether the genetic distance between viral isolates was associated with their sampling location and the origin of chickens (i.e. the LBM that chickens were sampled in, the LBM/farm type that poultry originated from or the province that poultry originated from). To do this, we utilized the 12 Vietnamese H9N2 viruses which we had full‐genome sequence data for and concatenated their open reading frames. The genetic distance between any two of the 12 fully sequenced H9N2 isolates decreased if these two isolates originated from the same LBM (Mantel test, *r* = −.41, *p = .004*), or sampled chickens were sourced in the same province (*r* = −.37, *p = .031*) (see Table S1 for genotype distribution between sampled LBM and province source). In the light of this, we were able to classify viruses into seven different sub‐genotypes using a > 98% nucleotide difference cut‐off for each gene segment (for viruses where full‐genome sequencing data were available) (Figure 2). From this, we could see that several strains which originated from the same LBM were also grouped into the same genotype; genotype VN4 contained three viruses from LBM Pho Hien, and genotype VN5 contained three viruses from LBM Do. HA and NP genes had the greatest maximum nucleotide pairwise distance with 6.7% and 6.9%, respectively, followed by NS with 5.7%, NA with 5%, PB1 with 4%, PB2 with 3.9%, M with 1.6% and PA with 1.5%.

#### 3.2.1 Molecular characteristics of virus isolates

All H9N2 viruses were low pathogenic avian influenza (LPAI) viruses due to the presence of a dibasic cleavage motif RSSR/G in the haemagglutinin (HA) glycoprotein. However, the partial sequencing data for the HA genes of the H5N6 viruses contained the polybasic cleavage motif RRKR/G, classifying them as highly pathogenic avian influenza (HPAI) viruses (Chen et al., 1998). All sequenced NA genes of the H9N2 viruses contained a three amino acid deletion between residues 62 and 64. Deletions in the stalk of NA are associated with adaptation of avian influenza viruses (AIVs) to chickens (Sorrell, Song, Song, Pena, & Perez, 2010); however, a functional balance between HA and NA must be maintained which may be reflected here by the retention of a NA stalk deletion, the continued HA1 glycosylation at residues 11, 123, 200 (6/16 sequenced HAs), 280, 287 and 295, and the receptor binding site residues A/T180, L216 and M217 (mature H9 numbering), which may have a variable impact on receptor binding (Baigent & McCauley, 2001; Castrucci & Kawaoka, 1993; Sealy et al., 2019). As previously reported, H9N2 viruses in Vietnam continue to retain the PB2 E627 amino acid and show no markers of resistance to neuraminidase inhibitors (Thuy et al., 2016).

#### 3.2.2 Risk factors associated with influenza virus A infection in chickens

Univariable analysis was used to identify potential risk factors related to the origin and management of poultry by traders, and subsequently included in multivariable analysis. Thirteen of 19 explanatory variables were identified as having a significant association with influenza virus A infection in chickens (Table 2): for example poultry being sold by retailers, sourced from other LBMs, sourced from middlemen, increased frequency of having unsold birds, having greater numbers of unsold ducks per day, storing unsold birds at home, increased number of days of trading in the LBM and had a strong positive association with influenza virus infection in chickens. Sourcing birds from large commercial farms and selling more chickens per day were negatively associated with influenza virus infection in chickens.

**Table 2:** Univariable analysis of potential risk factors for Influenza virus A infection in chickens

Variable	Response level	Median (Range)	Observations (%)	Influenza A positive	Influenza A negative	Odds Ratio (OR)	CI_95_ for OR	*P*‐value
Time spent at LBM	Numerical (hours)	5 (1–24)		168	318	0.96	0.91–1.00	.07
Target Buyer	Both		170 (35)	31	139	1		
	Consumer		209 (43)	125	82	6.84	4.28–11.17	<.001
	Vendor		107 (22)	12	97	0.55	0.26–1.10	.15
Number of different sources	Numerical	1 (1–4)		168	318	1.53	0.87–2.70	.13
Sourced from backyard farm (<50 birds)	Yes		112 (23)	40	71	1.09	0.69–1.68	.71
	No		374 (77)	128	247	1		
Sourced from small commercial farm (50–500 birds)	Yes		102 (21)	29	71	0.73	0.44–1.16	.19
	No		384 (79)	139	247	1		
Sourced from large commercial farm (>500 birds)	Yes		160 (33)	17	141	0.14	0.07–0.23	<.001
	No		325 (67)	151	177	1		
Sourced from another LBM	Yes		58 (12)	38	18	4.87	2.71–9.03	<.001
	No		428 (88)	130	300	1		
Sourced by a middleman	Yes		97 (20)	53	43	2.95	1.86–4.67	<.001
	No		389 (80)	115	275	1		
Chickens sold/day	Numerical	30 (1–2,400)		168	318	0.39	0.31–0.47	<.001
Ducks Sold/day	Numerical	0 (0–60)		168	318	1.07	1.03–1.09	<.001
Pigeons sold/day	Numerical	0 (0–100)		168	318	1.15	1.07–1.27	<.001
Days with unsold birds	Numerical	4 (0–7)		168	318	1.43	1.32–1.55	<0.001
Chickens unsold/day	Numerical	7 (0–1,000)		168	318	0.99	0.98–0.99	.01
Ducks unsold/day	Numerical	0 (0–30)		168	318	1.32	1.21–1.45	<.001
Storage location of unsold birds	All birds sold		78 (16)	10	70	1		
	Home		364 (75)	157	209	5.25	2.74–11.15	<.001
	LBM		44 (9)	1	39	0.18	0.009–0.98	.09
Resupply frequency	Every two days		39 (8)	16	24	1		
	Everyday		345 (71)	111	236	0.67	0.34–1.24	.31
	≤3 days/week		102 (21)	41	58	1.06	0.50–2.26	.88
Vaccination status	No		199 (41)	82	117	1		
	Yes		287 (59)	86	201	0.61	0.41–0.89	.01
Number of LBMs visited/week	Numerical	1 (1–5)		168	318	1.19	0.77–1.81	.41
Number of visits to current LBM/week	Numerical	7 (1–7)		168	318	1.80	1.46–2.24	<.001

Note

The total number of samples used in univariable and multivariable analyses was 486 after samples with incomplete questionnaires were removed.

In the final multivariable model, the sampled LBM was used as a random effect because poultry traders were naturally grouped into the eight selected LBMs. Three risk factors and one protective factor were identified. The risk factors included sourcing poultry from middlemen, selling poultry to consumers and having a greater number of ducks unsold per day (Table 3). The protective factor was selling more chickens per day.

**Table 3:** Multivariable analysis identifying risk factors for influenza virus A infection in chickens

Potential risk factors	Odds Ratio (OR)	CI_95_ for OR	*P*‐value
Selling only to consumers	2.72	1.52–4.84	<.001
Buying from middlemen	2.05	1.14–3.66	.02
Number of chickens sold/day	0.48	0.24–0.97	.04
Number of ducks unsold/day	1.33	1.02–1.78	.03

#### 3.2.3 Summary of poultry vendor practices

To put the identified risk factors into a broader context, we summarized poultry trading practices that were associated with the identified risks. Vendors who reported sourcing their birds from large commercial farms also sold a relatively large volume of chickens, with a median of 200 (IQR = 434) chickens sold per day. These vendors also primarily sold to other vendors (selling to vendors = 75, consumers = 14 and both = 69). In contrast, vendors who reported sourcing their birds from middlemen sold a relatively small volume of chickens, with a median of 15 (IQR = 20) chickens sold per day. These vendors were also seen to primarily sell directly to consumers (selling to vendors = 9, consumers = 60 and both = 27).

## 4 DISCUSSION

In our study, we have shown the G57‐like genotype of LPAI H9N2 viruses continues to co‐circulate with HPAI H5 viruses in Vietnam. We show there is reduced virus diversity between viruses from the same LBM and from the same province as compared to viruses from different LBMs and different provinces. This may indicate that populations of viruses that are genetically distinct are present within discrete parts of the poultry trade network. We also showed that trade practices influence the risks of influenza virus A detections in chickens. Given that H9N2 and H5Nx viruses are co‐circulating, risk mitigation strategies are likely to be effective against multiple subtypes.

A previous study by Fournié et al. (2012) have shown that it is possible to identify specific and distinct trader profiles of LBM sellers in Vietnam. As such, traders are classified as retailers or wholesalers based on who they primarily sell poultry to; retailers primarily sell directly to consumers, whereas wholesalers primarily sell to other poultry vendors within the trading network. In our study, we show retailers experienced higher odds of infection due to their trading practices. The retailers in our study were those who sourced their birds from middlemen, sold a relatively small volume of chickens and primarily sold directly to consumers. The risk factors associated with influenza virus A infection in chickens, selling only to consumers and buying from middlemen, can therefore be linked to retailers, which highlights their potential role in disseminating virus through the poultry trade network. In contrast, the wholesalers in our study were those who sourced from large commercial farms, sold a large volume of chickens and primarily sold to other vendors. The protective risk factor of selling more chickens is associated with the practices of wholesalers and identifies this group of poultry traders as relatively low risk.

When considering the potential impact on AIV dissemination that these traders can have, it is important to take note of the position that vendors have in the poultry trade network. Vendors who have strong connections to a network of contacts operating in and around LBMs would be expected to have a more pronounced role in disseminating AIVs, whereas vendors holding a loose link to a network of contacts may have a reduced impact on AIV dissemination (Fournié et al., 2016). Thus, middlemen are mobile, highly connected poultry traders that travel between farms and LBMs to purchase and sell birds, mixing poultry from many different sources. As a consequence, they facilitate a network of LBMs that are tractable to the circulation of influenza viruses (Fournié et al., 2013, 2012). The identification of middlemen supplying poultry to traders as a risk factor for influenza virus infection could be explained by their mobility and propensity to mix poultry, and their high connectivity to the poultry trade network. Likewise, retailers could be associated with higher odds of infection because they may purchase birds that have “changed hands” multiple times, promoting the amount of time spent by birds within the trade network and facilitating the mixing of birds from different sources. All the LBMs included in this study were open seven days a week, which would allow for greater connectivity between traders as they have more opportunity to interact at LBMs, potentially increasing the risk posed by retailers in particular. Although we did not explicitly capture the structure of the trade network in our study, the trading practices that we assessed can be used as indicators for the position of traders within the trade network.

In Vietnam, outbreaks of highly pathogenic avian influenza in spatially dispersed communes were shown to be closely linked to practices in agri‐livestock farming systems, which involve communities producing rice, and domestic aquatic birds and chickens (Pfeiffer, Minh, Minh, Martin, Epprecht, & Otte, 2007). These systems necessitate the use of areas with surface water such as river deltas, and therefore introduce the risk of mixing wild aquatic birds with domestic aquatic birds and chickens. Duck farming often involves raising and storing ducks in open bodies of water, which introduces the risk for wild waterfowl to mix with farmed ducks and transmit influenza viruses. In addition, studies have shown longer virus shedding times for LPAI‐infected ducks, up to 11.5 days, compared with LPAI‐infected chickens, up to 6 days (Hénaux & Samuel, 2011; James et al., 2016). Therefore, poultry traders with larger numbers of unsold ducks could increase the transmission window for ducks to infect chickens, especially as unsold ducks may have repeated exposures to wild waterfowl when traders store unsold ducks at home.

Effective control of avian influenza requires understanding risk factors associated with contamination of all aspects of poultry production. Contamination of the environment in LBMs and of utensils used for handling live and slaughtered poultry has been well documented; risk factors associated with environmental contamination of LBMs include “in‐house” poultry slaughtering, and their location in regions which see great chicken density and poultry‐related activity (Indriani et al., 2010). Avian influenza viruses are frequently detected in shared poultry water (Leung et al., 2007), wooden tabletops, cages, bins and floors (Indriani et al., 2010). In our study, we have confirmed the importance of environmental contamination by showing influenza virus A prevalence in three areas of LBMs: slaughter area, waste area and poultry storage area. Traders who bring infected birds to LBMs play a role in perpetuating environmental contamination, while traders with healthy birds run the risk of contaminating their birds by storing them in contaminated environments.

Analysis of the N2NA amino acid sequences revealed a stalk deletion is present in all viruses, highlighting the sustained poultry adaptation of H9N2 AIVs in Vietnam (Sorrell et al., 2010, Thuy et al., 2016). However, amino acid diversity at residue 180 of the HA protein could play an important role in zoonotic potential. Previously, we and others have shown that H9N2 viruses carrying the A180T/V substitution gain the ability to bind to human‐like receptor analogues (Sealy et al., 2019; Teng et al., 2016; Yang et al., 2017). The A180T/V substitution also enhances binding avidity towards avian‐like receptor analogues, which can attenuate virus replication in vitro*;* however, the impact of this mutation in conjunction with a NA stalk deletion is currently unknown.

Finally, vaccination against H9N2 has not been adopted in Vietnam; however, vaccination programmes against H5 are a key component of outbreak response measures (Nguyen et al., 2014). Both large commercial farms and backyard flocks are included in emergency response H5 vaccination programmes (Domenech et al., 2009), and discretionary use of routine anti‐H5 vaccines is practiced within some commercial farms in provinces believed to be high risk. Going forward, vaccination in farms in highly connected trade networks where high‐risk traders operate, as identified in this study, may be beneficial in mitigating AIV dissemination.

The primary limitation to our study was that poultry and poultry traders may have been repeatedly sampled and questioned during our repeated visits to each LBM. We did not record who we had included in our study during the seven‐day sampling periods at each LBM, which meant that if a vendor had unsold chickens from a previous day then we could have sampled those birds multiple times. Likewise, we may have received feedback on poultry trading practices from the same vendors multiple times over the sampling period. However, the infection status of unsold chickens and associated poultry trading practices of a vendor may have changed as the week progressed, that is chickens may have been free of AIV infection at the start of the sampling week, but by day 2 or day 3, the chickens of the repeatedly sampled poultry trader may have become AIV‐positive. This may be reflected by the repeatedly sampled poultry trader having more unsold birds compared to earlier in the week.

In conclusion, we have identified poultry trade practices that impact the risk of influenza virus A infection in chickens, and we have been able to attribute these practices to certain types of poultry trader. Being able to identify a specific type of poultry trader responsible for impacting AIV dissemination due to their poultry trading practices is novel and could be useful in future surveillance and control programmes. H9N2 viruses continue to cause significant poultry outbreaks and expand their global distribution within poultry producing countries. It is therefore increasingly important to monitor trends in H9N2 epidemiology, by using both active and passive surveillance systems that are already in place for H5 pandemic preparedness. Surveillance of AIVs is particularly important in countries where there is co‐circulation of multiple subtypes. Prevention and control of zoonotic risks associated with endemic AIVs require continued surveillance efforts, and cost‐effective targeted approaches to identify and protect high‐risk poultry traders in highly connected trade networks.

## ETHICS STATEMENT

The approval for the study was obtained from the Department of Animal Health (DAH), Hanoi, and from each sub‐department (SDAH) with jurisdiction over the provinces where selected LBMs were situated. FAO‐supported surveillance of avian influenza viruses in LBMs is routinely conducted in northern Vietnam by NCVD and DAH, Hanoi, who enabled the implementation of this study.

## CONFLICT OF INTERESTS

The authors declare no competing interests.

## AUTHOR CONTRIBUTIONS

JES, GF and JEB conceptualized the data. JES and GF involved in formal analysis. MI acquired funding. JES, PHT and NHD investigated the data. JES and GF provided methodology, resources and software. GF, HRvD, JEB and MI supervised the data. JES wrote the original manuscript. All reviewed and editing the manuscript.
