# Supplementary material for: Poultry trading behaviours in Vietnamese live bird markets as risk factors for avian influenza infection in chickens
Source: Transbound Emerg Dis. 2019 Aug 9;66(6):2507–16. doi: 10.1111/tbed.13308 (PMC6899644; doi:10.1111/tbed.13308)
Supplement: Supplementary file 1 [file TBED-66-2507-s001.docx]

Poultry trading behaviours in Vietnamese live bird markets as risk factors for avian influenza infection in chickens

Joshua E. Sealy^1,2^, Guillaume Fournie^2^, Pham Hong Trang^3^, Nguyen Hoang Dang^3^, Jean-Remy Sadeyen^1^, To Long Thanh^3,4^, H. Rogier van Doorn^5^, Juliet E. Bryant^6^, Munir Iqbal^#1^

**Supplementary data**

**Table S1. Distribution of virus genotypes**

Virus isolates from poultry traders who were unable to recall which province their chickens came from were not included in mantel tests that involved testing province source.

**Figure S1. Phylogenetic trees of H9N2 viruses**


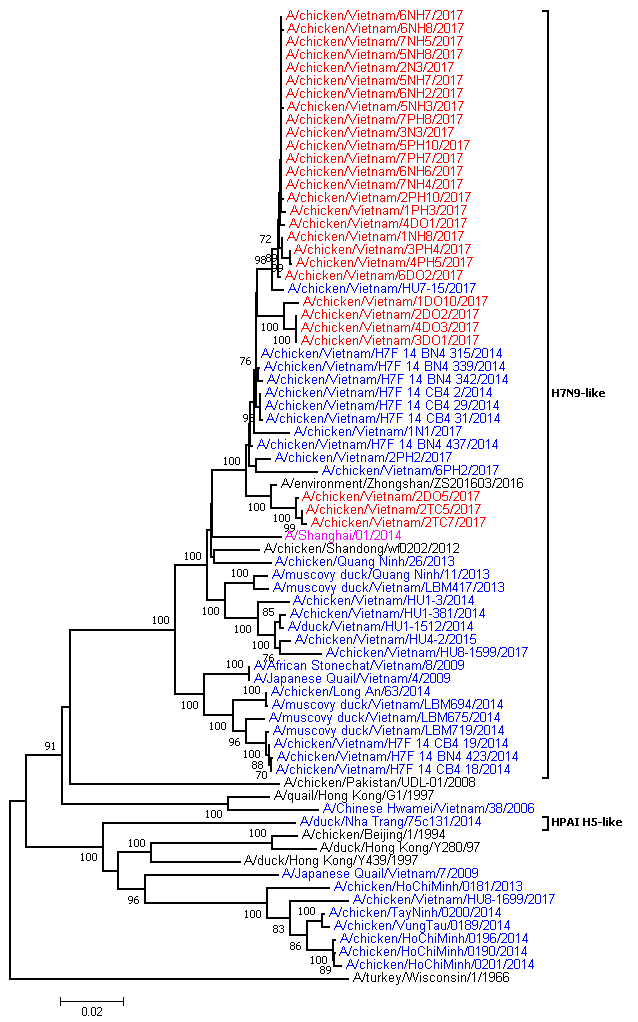


**PB2**


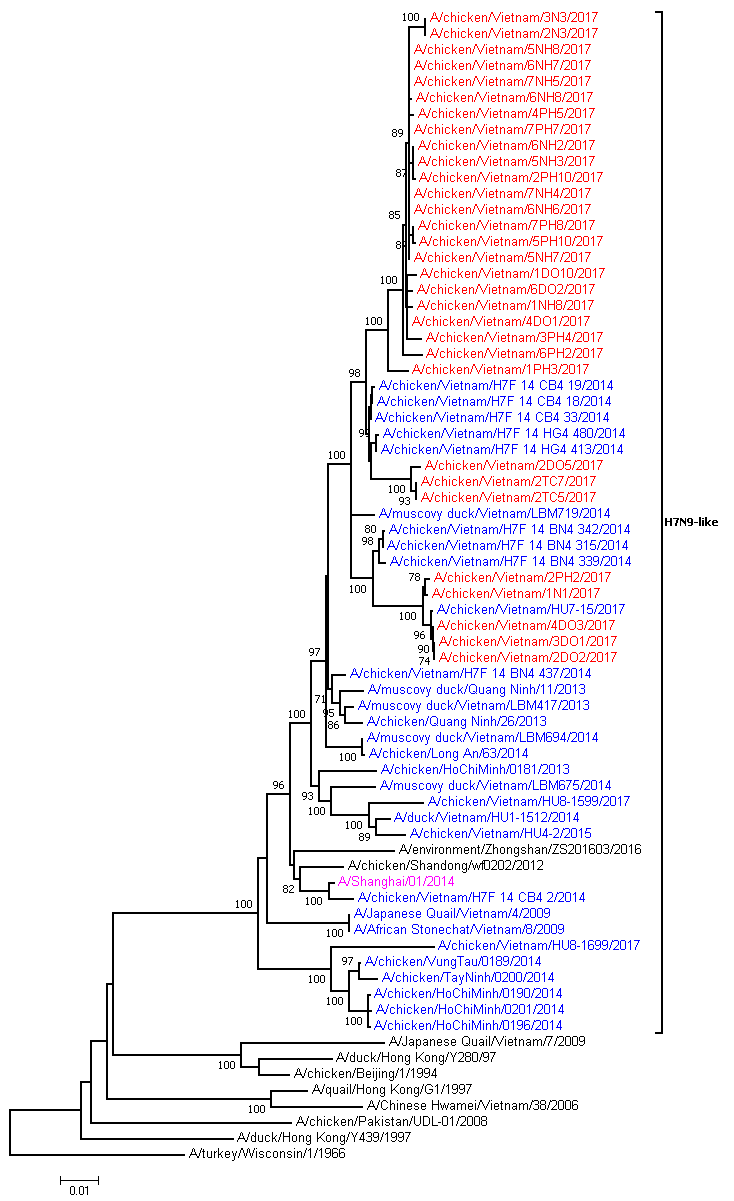


**PB1**


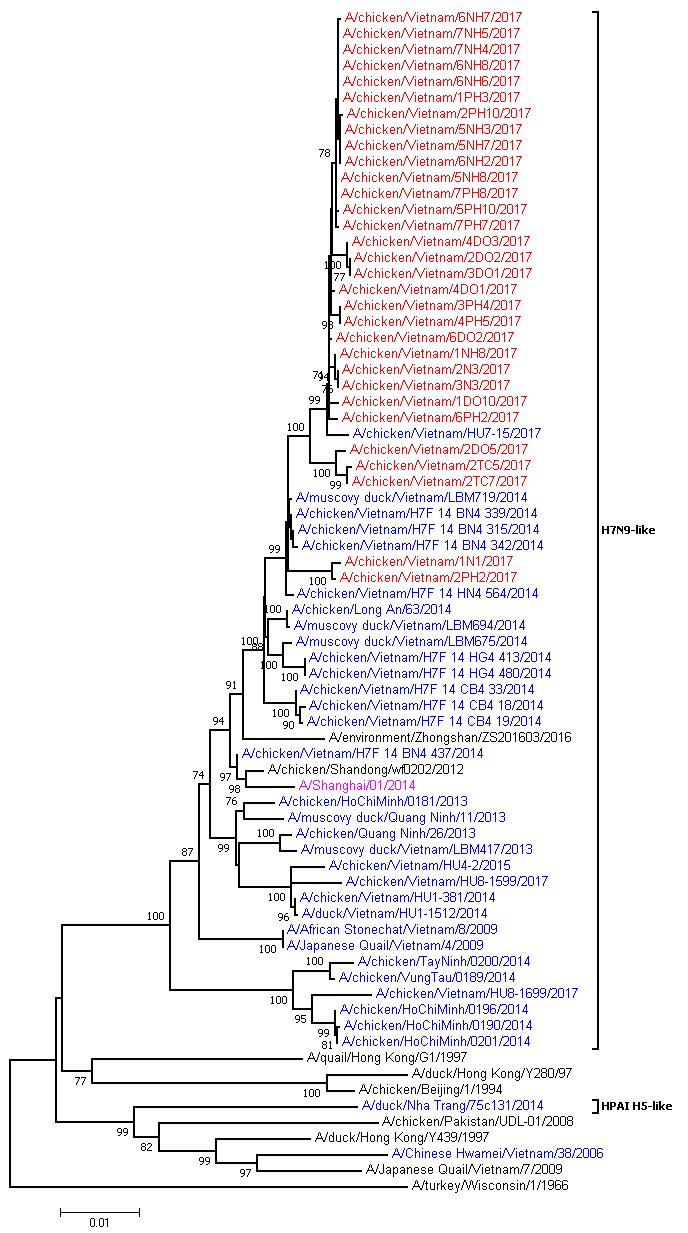


**PA**

**NP**


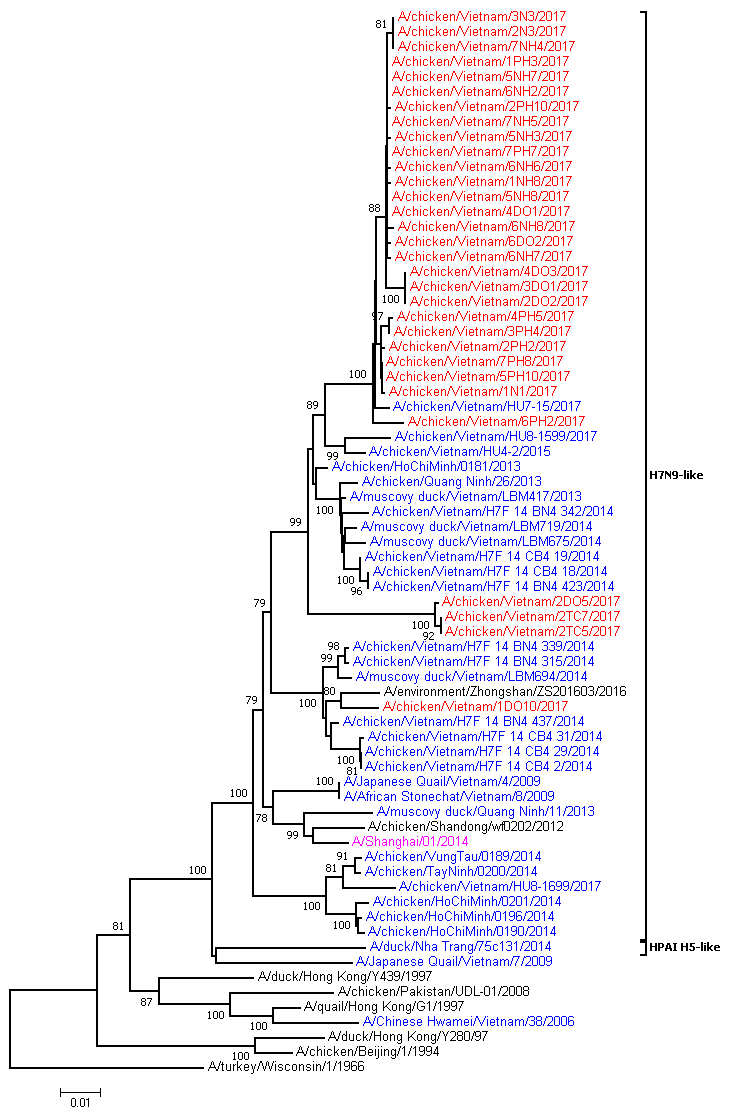

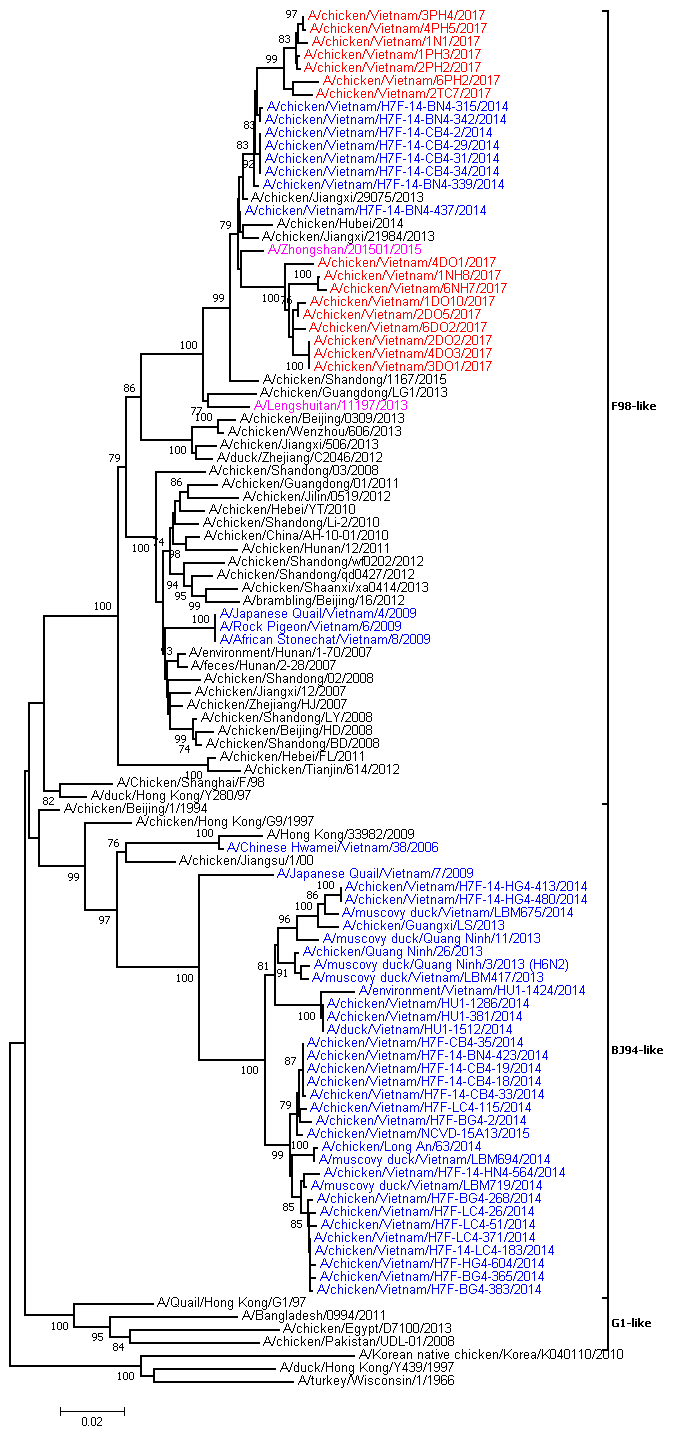


**NA**


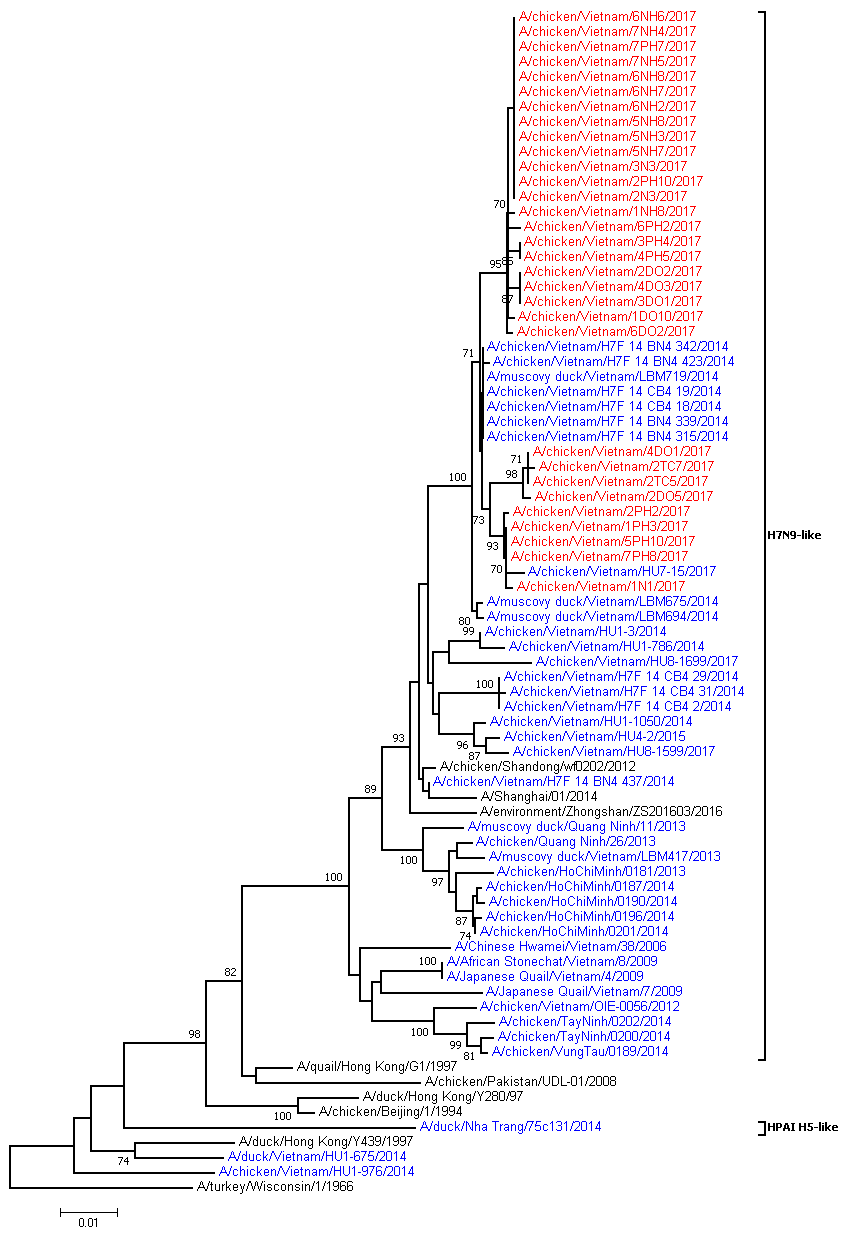


**MP**


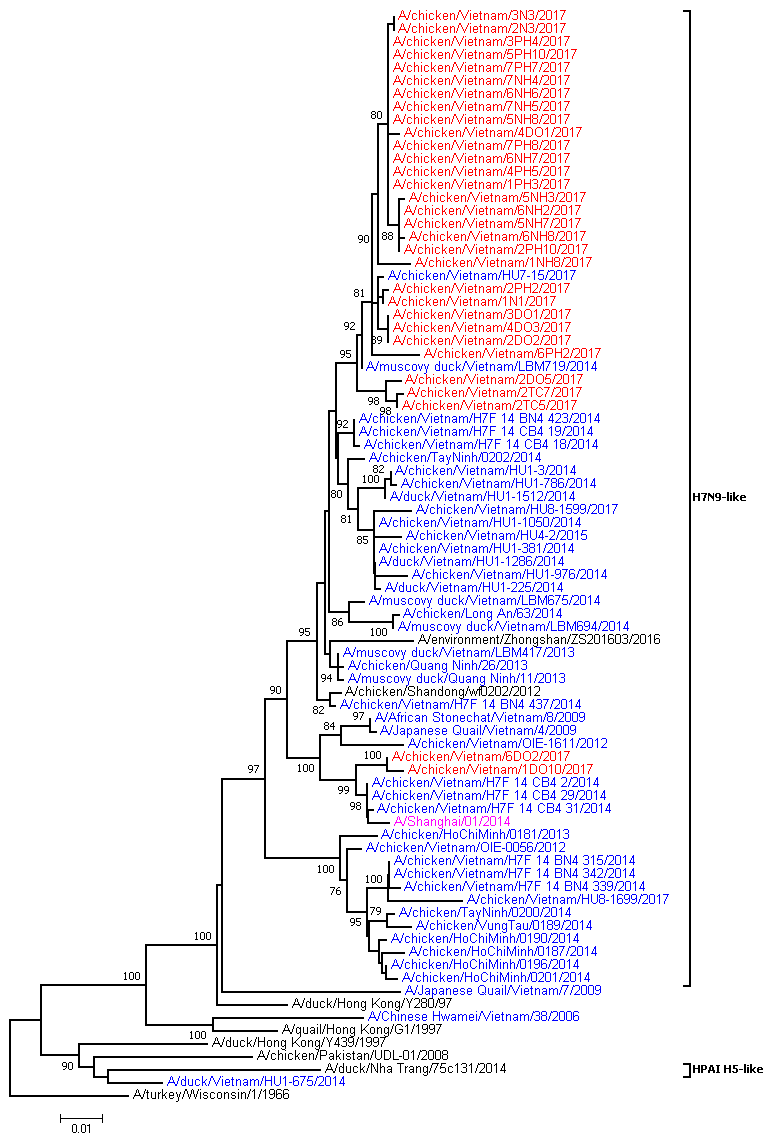


**NS**

Neighbour joining trees representing phylogeny of H9N2 sequences generated in this study; reference strains from NCBI and GISAID databases are included for comparison. Trees were formed with 1000 bootstrap replicates, bootstrap values <70 are not shown. In blue are reference Vietnam strains, in red are strains from this study, in black are non-Vietnam strains, and in fuchsia are recent human isolates.

**Questionnaire**

| Vietnamese translation here \| How long will you spend in this market? Answer in hours | Vietnamese translation here \| Who do you intend on selling your poultry to? | |
| --- | --- | --- |
|  | Consumer |  |
|  | Vendor |  |
|  | Other (describe) |  |

| VN translation here \| Where did you get your poultry from?  Record province, district and farm/market name. If the flock is made up of poultry from more than one place then record each place. | VN translation here \| What type of poultry production system do your poultry come from?  Tick the boxes and note that more than one box can be ticked if poultry come from more than one source. Include farm/market name if possible. | |
| --- | --- | --- |
|  | Farm – backyard (<50 birds) |  |
|  | Farm – small commercial (50-500) |  |
|  | Farm – large commercial (>500) |  |
|  | Another live bird market – include name |  |
|  | Other (describe) |  |

| VN translation here \| What are the poultry species/breeds that you sell? (Please tick the species and describe the breed) | | VN translation here \| How many birds of each species do you sell in a day? |
| --- | --- | --- |
| Chicken (broiler/layer/native/other) |  |  |
| Duck (meat/layer/Muscovy/other) |  |  |
| Pigeon |  |  |
| Other (describe) |  |  |

| VN translation here \| From the past week, how many days have you had unsold poultry?  Answer should be between 0 and 7 days | VN translation here \| On the days that you have unsold poultry, what number of each bird species remain unsold? Include breed of each species if it is known | | VN translation here \| On the days that you have unsold poultry, where do you store your unsold poultry? | |
| --- | --- | --- | --- | --- |
|  | Chicken |  | Home |  |
|  | Duck |  | Live bird market |  |
|  | Pigeon |  | Other (Describe) |  |
|  | Other |  |  |  |

| VN translation here \| How often do you re-supply your stock of poultry? | | VN translation here \| Are the birds in your flock vaccinated against influenza?  A yes/no/don’t know answer |
| --- | --- | --- |
| Everyday |  |  |
| Every 2 days |  |  |
| Three or less times a week |  |  |

| VN translation here \| How many different markets will you visit in a week? | VN translation here \| How many times will you visit this market in a week? |
| --- | --- |
|  |  |
